# Toll-Like Receptor 4 Mutant and Null Mice Retain Morphine-Induced Tolerance, Hyperalgesia, and Physical Dependence

**DOI:** 10.1371/journal.pone.0097361

**Published:** 2014-05-13

**Authors:** Theresa Alexandra Mattioli, Heather Leduc-Pessah, Graham Skelhorne-Gross, Christopher J. B. Nicol, Brian Milne, Tuan Trang, Catherine M. Cahill

**Affiliations:** 1 Department of Biomedical and Molecular Sciences, Queen’s University, Kingston, Ontario, Canada; 2 Departments of Comparative Biology & Experimental Medicine, Physiology & Pharmacology, Hotchkiss Brain Institute, University of Calgary, Calgary, Alberta, Canada; 3 Department of Pathology and Molecular Medicine, Queen’s University, Kingston, Ontario, Canada; 4 Cancer Biology and Genetics Division, Cancer Research Institute, Queen’s University, Kingston, Ontario, Canada; 5 Department of Anaesthesiology & Perioperative Medicine, Queen’s University, Kingston, Ontario, Canada; 6 Department of Anesthesiology and Perioperative Care, University of California Irvine, Irvine, California, United States of America; University of Kentucky Medical Center, United States of America

## Abstract

The innate immune system modulates opioid-induced effects within the central nervous system and one target that has received considerable attention is the toll-like receptor 4 (TLR4). Here, we examined the contribution of TLR4 in the development of morphine tolerance, hyperalgesia, and physical dependence in two inbred mouse strains: C3H/HeJ mice which have a dominant negative point mutation in the *Tlr4* gene rendering the receptor non-functional, and B10ScNJ mice which are TLR4 null mutants. We found that neither acute antinociceptive response to a single dose of morphine, nor the development of analgesic tolerance to repeated morphine treatment, was affected by TLR4 genotype. Likewise, opioid induced hyperalgesia and opioid physical dependence (assessed by naloxone precipitated withdrawal) were not altered in TLR4 mutant or null mice. We also examined the behavioural consequence of two stereoisomers of naloxone: (−) naloxone, an opioid receptor antagonist, and (+) naloxone, a purported antagonist of TLR4. Both stereoisomers of naloxone suppressed opioid induced hyperalgesia in wild-type control, TLR4 mutant, and TLR4 null mice. Collectively, our data suggest that TLR4 is not required for opioid-induced analgesic tolerance, hyperalgesia, or physical dependence.

## Introduction

Morphine and related opioids are among the most potent and widely prescribed drugs for treating moderate to severe pain. Their hallmark analgesia is predominantly mediated by activation of G_i/o_ protein-coupled mu opioid receptors [1], [2], which are expressed throughout the central nervous system. Within the dorsal spinal cord, mu opioid receptors are found on pre- and post-synaptic nociceptive neurons, as well as on astrocytes and microglia [3]–[8]. Unlike neuronal mu opioid receptors, which are well characterized, the importance of mu opioid receptors on non-neuronal cells remains poorly understood. Growing evidence suggests mu opioid receptors expressed on microglia are causally implicated in the sequelae of opioid analgesic tolerance, physical dependence, and paradoxical pain (opioid induced hyperalgesia) [9]–[13]. These negative side effects are major barriers that limit the effective management of pain with opioid drugs.

Recent lines of evidence suggest glia are involved in opioid tolerance and hyperalgesia. In particular, it has been reported that glial inhibitors, such as fluorocitrate and propentofylline, prevent the development of, and reverse established, opioid analgesic tolerance in animal models [14]–[16]. Hutchinson and colleagues pioneered studies demonstrating that morphine and other opioid agonists have off-target effects at toll-like receptor 4 (TLR4) receptors [17], [18], which are widely expressed in spinal microglia, macrophages [19], [20], and astrocytes [21], [22]–[24]. Toll-like receptors are single transmembrane receptors that recognize a variety of endogenous (e.g. heat shock proteins) and exogenous (e.g. lipopolysaccharides; LPS) substances that signal danger and initiate immune responses [17], [21], [25]. However, unlike opioid receptors, which are selective for the (−) isomers of opioids, TLRs can bind either (+) or (−) isomers of opioid ligands [17],[26]. Activation of TLR4 induces significant gliosis and is implicated in opioid tolerance, hyperalgesia, physical dependence, respiratory depression and addiction [25], [27]–[31]. A role for TLR4 in opioid action was surmised based on reports that acute morphine analgesia is potentiated in TLR4 null mutant mice, and that (+)naloxone, a purported TLR4 antagonist, prevents morphine tolerance, hyperalgesia, and opioid reinforcement.

Since the first demonstration that the inactive opioid receptor stereoisomer of naloxone binds TLR4, there has been considerable interest in the clinical translation of its use to improve opioid analgesia. Along these lines, we recently reported that ultra-low dose (picomolar to nanomolar) naltrexone attenuates the development of morphine tolerance and suppresses morphine-induced spinal gliosis [32]. Importantly, we showed that morphine-induced hyperalgesia, but not tolerance, is attenuated by ultra-low doses of (+)naloxone [13]. However, in contrast to the reports suggesting TLR4 is the target for (+)naloxone-induced effects, we reported that (+)naloxone remained effective in blocking morphine-induced hyperalgesia in TLR4 deficient mice [13]. The present study examined the role of TLR4 in the development of morphine-induced analgesia, analgesic tolerance, hyperalgesia, and physical dependence using TLR4 null and TLR4 mutant mice. Additionally, it was of interest to reaffirm whether TLR4 is the target for how ultra-low dose (+)naloxone attenuates opioid-induced gliosis and morphine-induced hyperalgesia.

## Methods

### Animals

Experiments were performed on naïve and morphine-treated adult male (8–10 weeks of age) mice. The following inbred strains were used, all obtained from the Jackson Laboratory (Bar Harbor, Maine, USA): TLR4 mutant C3H/HeJ and C3H/HeN or C3H/HeOuJ (controls), C57BL/10ScNJ (hereinafter called B10ScNJ) and C57BL/10ScSNJ (hereinafter called B10ScSNJ – control). Mutant TLR4 (C3H/HeJ) mice possess a missense mutation (A→C) of the TLR4 gene at position 2342 of the cDNA sequence resulting in a substitution of histidine for proline, thus producing a non-functional TLR4-protein and conferring resistance to LPS [33],[34]; C3H/HeOuJ are the closest wild type substrain [35]. The B10ScNJ strain has a *Tlr4Lps-del* spontaneous mutation that completely removes the *Tlr4* coding sequence. No TLR4 mRNA or protein is expressed and is equivalent to a TLR4 knockout mouse. Homozygous mutants exhibit a defective response to LPS stimulation and the functionality of these mice is similar to the *Tlr4Lps-d* mutation found in C3H/HeJ mice [34]; the B10ScSNJ strain is the closest to a wild type strain. Mice were housed 3–4 in standard shoe box cages with *ad libitum* access to food and water, and maintained in a temperature-controlled (22±1°C) environment on a 12/12 h light/dark cycle with lights on at 7∶00 h. Animals were housed for at least 1 week prior to testing, and habituated to testing environment for 4–5 days in order to reduce stress-related analgesia.

### Ethics Statement

All experimental protocols were approved by the Queen’s University and University of Calgary Animal Care Committees, and complied with the policies and directives of the Canadian Council on Animal Care and the International Association for the Study of Pain.

### Drug Treatments

Morphine sulphate was purchased from Sabex, Kingston General Hospital, Kingston, Ontario, Canada or PCCA Corp., London, Ontario, Canada. Levo (−)naloxone (hydrochloride) and lipopolysaccharide from Escherichia coli O111: B4 were purchased from Sigma-Aldrich (St. Louis, MO, USA). Dextro (+)naloxone (hydrochloride) was a kind gift of the National Institute on Drug Abuse. All drugs were prepared in 0.9% sterile saline solution and administered via intraperitoneal (i.p.) injection.

### Thermal Nociception

The effect of drug administration on thermal nociceptive responses was assessed using the tail flick assay. Briefly, the distal portion of the tail is immersed in hot water (50°C) and the latency to a vigorous tail flick was measured. Three baseline latencies were measured prior to drug injection to determine the normal nociceptive responses of the animals. A cut-off time of 10 s was imposed to avoid tissue damage. Tail-flick latencies were converted to a maximum possible effect (% MPE): (post-drug latency − baseline)÷(cut-off latency − bbaseline)×100. All behavioural testing was performed by an experimenter blinded to drug treatment.

### Mechanical Withdrawal Thresholds

The effect of drug administration on mechanical nociceptive responses was assessed by measuring withdrawal responses to von Frey filament stimulation. Briefly, animals were placed in Plexiglas boxes on a wire grid through which a 0.4 g von Frey filament was applied to the plantar surface of the hind paw. The number of paw withdrawals to 10 repeated stimulations was recorded for each animal prior to drug administration (before morning injection) or 24 h post-treatment. All behavioural testing was performed by an experimenter blinded to drug treatment.

### Gene Expression by Quantitative Real-time Polymerase Chain Reaction

Mice were sacrificed by decapitation under light halothane anesthesia and their spinal cords were immediately removed by spinal ejection. Total RNA was isolated, using the TRIZOL method, from the dorsal lumbar spinal cord (approximately the L4/L5 level). Spectrophotometric analysis was performed using a Biotek Synergy HT plate reader to monitor RNA purity and concentration and only RNA with a 260 nm/280 nm ratio of 1.8 to 2.0 was considered acceptable. RNA samples were then converted to cDNA using the iScript cDNA synthesis kit (Bio-Rad). cDNA was quantified and assessed similar to RNA samples. cDNA was combined with iQ SYBR Green mix (Bio-Rad), autoclaved water and the necessary primers according to protocol to evaluate expression of CD11b, GFAP and GAPDH. Primers were purchased from Invitrogen (San Diego, CA), with the following sequences: CD11bF, CAG ATC AAC AAT GTG ACC GTA TGG G and CD11bR,CAT CAT GTC CTT GTA CTG CCG CTT G [18]; GFAPF, GAT CGC CAC CTA CAG GAA AT and GFAPR, GTT TCT CGG ATC TGG AGG TT [36]; mGAPDHF, TCA TGA CCA CAG TGG ATG CC and mGAPDHR, GGA GTT GCT GTT GAA GTC GC. The Bio-Rad thermocycler was programmed to perform a 5 min 95°C hot-start followed by 50 cycles of 95°C for 15 s, 65°C for 15 s and 72°C for 30 s. Annealing temperature was optimized using primer efficiency curves for all primer pairs using a 7-point 2x dilution series of cDNA pooled from control samples. All primer efficiencies were in the acceptable range of 90–100%.

### Immunohistochemistry

#### Iba-1 immunofluorescence

Mice (n = 5–6 per group) were anesthetized with sodium pentobarbital (70 mg/kg, i.p.) and perfused through the aortic arch with 50 mL of ice-cold 0.1 M phosphate buffered saline (PBS) followed by a freshly prepared solution of 4% PFA in 0.1 M phosphate buffer (PB) (60 mL, pH 7.4) at 4°C. Spinal cords were removed rapidly by hydrostatic propulsion. Tissue was then post-fixed in the same fixative solution by immersion for 48 h followed by cryoprotection in 30% sucrose in 0.2 M PB, both at 4°C, prior to embedding in OCT medium and stored at −80°C until sectioning. Immunohistochemistry was performed on lumbar spinal cord free-floating sections as used previously in our laboratory [13], [32], [37]. Briefly, 30 µm transverse sections were cut on a freezing microtome, washed with 0.1 M PBS, pH 7.4, followed by pre-incubation for 1 hour in a blocking solution containing 2% NGS and 0.3% Triton-X 100 at room temperature (RT). Serial sections were then incubated at 4°C for 24 hours with Iba-1 antisera (1∶500; Wako, WEG2172) in PBS with 2% NGS and 0.3% Triton-X 100. After rinsing with PBS (3×10 minutes), the sections were incubated at RT for 1 h in fluorescently-conjugated secondary antibodies (1∶1500; Cy3 donkey-anti-rabbit, Jackson Immuno Research). Sections were then washed in PBS (3×10 minutes) followed by a final wash containing DAPI (1∶10000) for 10 minutes. Sections were washed with water to remove salts and mounted on superfrost slides, air dried and cover-slipped with fluoromount (Sigma-Aldrich, F4680). Immunoreactive cells were visualized in the deep dorsal horn using a Nikon Eclipse Ti (C1SI Spectral Confocal) microscope 20x magnification lens (Scale bar = 100 µm) and images acquired and digitalized using E2-C1 software and converted using Nis Elements imaging software for quantitative analysis with Image J Software. Four images from 10 spinal cord sections per animal were captured per mouse for n = 5 mice per experimental group. Statistical analyses were performed using Prism 6.0 (Graph Pad, San Diego, CA). The mean intensity for each photomicrograph within the superficial dorsal horn of the spinal cord for each treatment group was calculated. Imaging and quantification were performed by an experimenter blind to genotype and treatment.

#### c-fos immunolabeling

Mice (n = 5–6 per group) were anesthetized with urethane and perfused intracardially with 0.1 M PB 4% paraformaldehyde (60 mL). Spinal cords were removed via hydrostatic propulsion with ice-cold saline using a 16-gauge blunted needle inserted into the sacral vertebral canal. The lumbar segment of the spinal cords was isolated and post-fixed overnight in 4% paraformaldehyde. Following 30% sucrose cyroprotection, spinal cords were sectioned at 30 µm thickness using a cryostat. Free-floating spinal cord sections were incubated with 0.3% H2O2 for 10 min and with 1% Sodium Borohydride for 5 minutes to block endogenous peroxidase and biotin activity. Immunohistochemistry was performed according to the avidin biotinylated-HRP complex (ABC) method using a standard ABC kit (Vector Laboratories, Burlingame, CA) as used previously in our laboratory [13], . Sections were incubated with rabbit anti-cFos antibody (1∶20,000; Calbiochem) diluted in 0.1 M PBS containing 0.3% Triton X-100 and 2% Normal Goat Serum for 24 h at 4°C. Following 1 h incubation with biotinylated anti-rabbit secondary antibody (1∶400; Vector Laboratories Inc., Burlingame, CA), sections were processed with Vectastain ABC kit (Vector Laboratories Inc.) and developed using 3,3-diaminobenzedine (Vector Laboratories Inc.) with nickel. After desiccation overnight, slides were desalted, dehydrated, defatted and cover-slipped using xylene-based medium. To quantify Fos activation, microscope images were captured using a Leica DM4000 B LED microscope (Leica Microsystems, Concord, ON). Representative images were acquired at 20x magnification lens (Scale bar = 100 µm). Spinal cord sections were randomly obtained from four B10ScNJ or B10ScSNJ mice treated with saline or morphine and challenged with naloxone. Fos-immunoreactive neurons were counted from images acquired at 20x using an Olympus Virtual Slide System Macro Slide Scanner (VS120-5 Slide). Counts were made of the total number of Fos-immunoreactive neurons within the spinal dorsal horn. The counts were averaged for each treatment group. Imaging and quantification were performed by an experimenter blind to genotype and treatment.

### Study Design

#### Acute Morphine-induced antinociception in control and TLR4 deficient mice

To evaluate the importance of TLR4 in acute morphine antinociception, control and TLR4 mutant (C3H/HeJ) and null (B10ScNJ) mice were administered a single injection of morphine (3 mg/kg, i.p.). Morphine antinociception was then assessed by the thermal tail-flick test at 15 minute intervals over 60 minutes post-injection. All responses were expressed relative to baseline tail-flick response, which was measured prior to the morphine injection.

#### Antinociceptive Tolerance to Chronic Morphine Treatment

To examine the role of TLR4 in the development of morphine analgesic tolerance, control and TLR4 mutant (C3H/HeJ) mice were administered morphine (10 mg/kg; i.p) once daily for 5 days. This dosing regimen was previously validated to produce robust opioid-induced analgesic tolerance [13]. Thermal nociception was assessed on days 1, 3 and 5 prior to drug injection and 30 minutes post-injection by tail flick test. Mice were euthanized 4 h following behavioural testing and spinal cord tissue collected for quantitative real-time PCR analysis to evaluate treatment effects on spinal microglia and astrocytes.

#### Opioid-induced hyperalgesia

To determine the involvement of TLR4 receptors in morphine-induced hyperalgesia, control and TLR4 mutant (C3H/HeJ) and null (B10ScNJ) mice were treated with morphine (day 1: 10 mg/kg i.p., day 2: 20 mg/kg i.p., day 3: 30 mg/kg i.p., days 4–7: 40 mg/kg i.p.) twice daily for 7 days. We have shown that this ascending morphine dosing paradigm produces robust and reliable opioid-induced hyperalgesia in mice. [13]. Mechanical withdrawal thresholds (described in section 2.3) were measured prior to the morning morphine injection each day (12 h after the last injection).

#### Effects of naloxone stereoisomers on morphine tolerance and hyperalgesia

To examine the stereoselective effects of naloxone on the development of analgesic tolerance and opioid induced hyperalgesia, we co-administered (+) naloxone or (−)naloxone with daily morphine. In the opioid tolerance studies (section 2.7.2), (+) or (−) naloxone was given with daily morphine over 5 days, whereas in the opioid induced hyperalgesia experiments (section 2.7.3). In both treatment paradigms, vehicle (saline) co-treatment with morphine was used as control.

#### Lipopolysaccharide-induced hyperalgesia

To assess the functionality of TLR4 receptors in TLR4 mutant mice, LPS (a TLR4 agonist) was used to induce mechanical hyperalgesia. Paw mechanical withdrawal thresholds (described in section 2.3) were measured prior to, and 24 h after, LPS (1 mg/kg, i.p.) injection in control, TLR4 mutant and TLR4 null mice.

#### Establishment of morphine physical dependence and behavioural assessment of naloxone-precipitated withdrawal

To assess the involvement of TLR4 receptors in physical withdrawal in opioid dependent animals, control (C3H/HeN and B10ScSNJ), TLR4 mutant (C3H/HeJ), and null (B10ScNJ) mice received intraperitoneal injections of systemic morphine at 8 h intervals (day 1: 7.5 and 15 mg/kg; day 2: 20 and 25 mg/kg; day 3: 30 and 35 mg/kg; day 4: 40 and 45 mg/kg). This dosing regimen for morphine administration was previously validated to produce robust naloxone precipitated withdrawal [13]. On day 5, mice received a morning injection of 50 mg/kg and 2 h later an injection of naloxone (2 mg/kg) to precipitate withdrawal. Control mice received saline and were challenged with naloxone on day 5. Mice were accli­matized to a clear Plexiglas testing chamber 1 h before naloxone. Signs of with­drawal were compiled as previously described [13]. Specifically, jumping, headshakes, wet-dog shakes and grooming behaviour were evaluated over 10 minute intervals for a total testing period of 30 minutes and assigned a standardized score of 0 to 3 (0 = absent; 1 = 1–3 bouts; 2 = 4–6 bouts; 3 = 7 bouts and greater). Paw tremors, piloerection, salivation and ejaculation were also evaluated, with one point being given to the presence of each sign during each 10-min interval. The number of periods showing the latter signs were then counted (maximum score of 3 per behavioural sign) and the scores were added together to yield a final cumulative withdrawal score. Mice were also weighed before and after naloxone challenge and weight loss (an indicator of micturition and defecation) was calculated. In all behavioural studies, experimenters were blind to the drug treatments and genotype of the mouse. Two hours after naloxone injection, animals were subjected to aldehyde perfusions for immunohistochemistry processing of c-fos and microglial activation in spinal cord tissue.

### Statistical Analyses

All values are expressed as the mean ± standard error of mean (s.e.m.). Statistical analyses were performed with GraphPad Prism 6.0 software (San Diego, CA, USA). Statistical significance for multiple group comparisons was determined by a one or two-way analysis of variance (ANOVA) followed by Tukey’s, Bonferroni’s or Dunnett’s *post-hoc* test or a 2-way ANOVA multiple comparisons test followed by Sidak’s post hoc analysis for comparing treatment and genotype. qRT-PCR data was analyzed by one-way repeated measures ANOVA followed by Tukey’s *post-hoc* test. A p-value less than 0.05 was considered significant.

## Results

### Acute Morphine-induced Antinociception in Control and TLR4 Deficient or Null Mice

**Figure 1:**
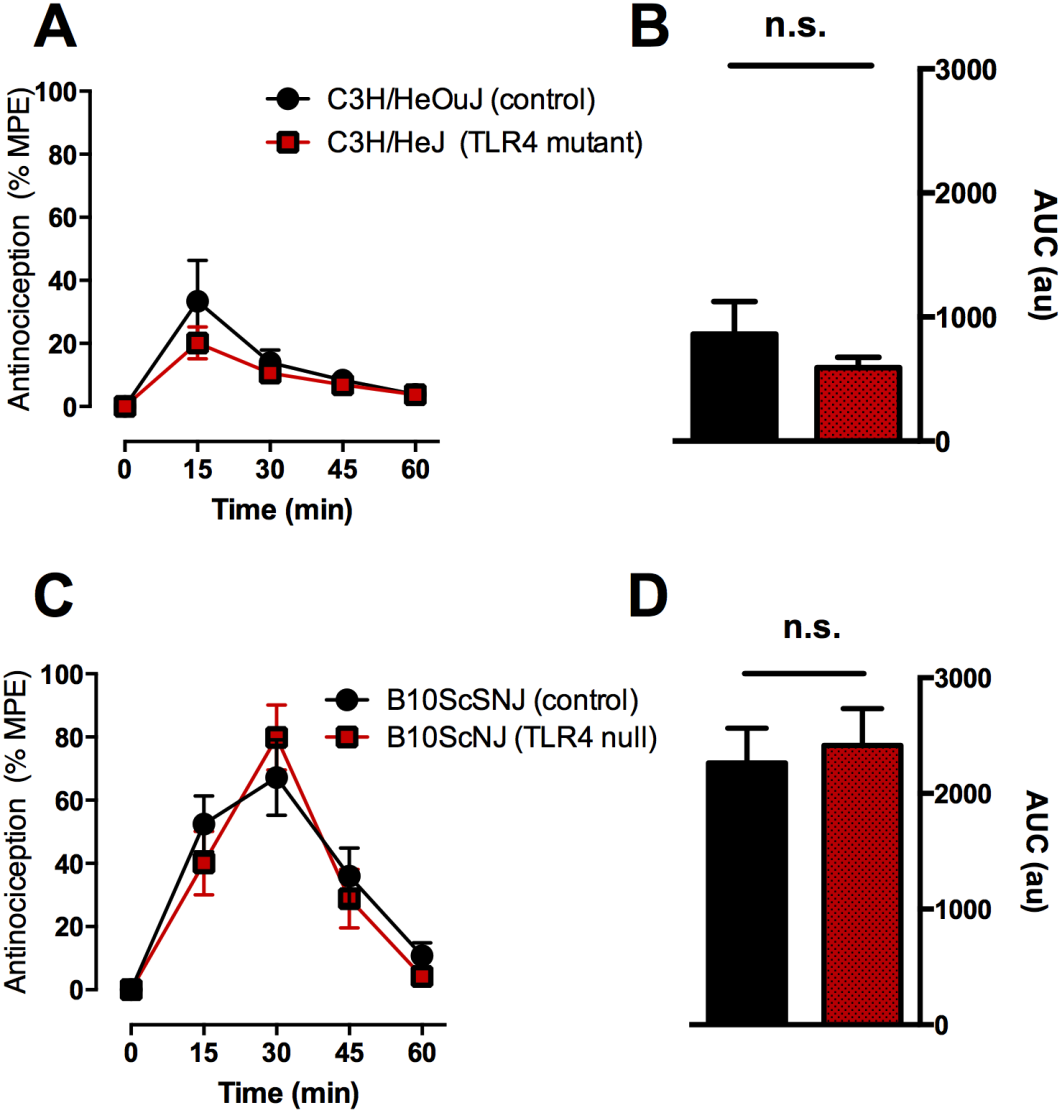
No difference in acute morphine-induced antinociception between in (A) C3H/HeJ TLR4 mutant or (C) B10ScNJ TLR4 null mice compared to their appropriate genotype/strain control. Animals were administered morphine (3 mg/kg, i.p.) and thermal nociceptive responses were measured every 15 minutes post-injection. No difference in antinociception was observed between genotypes at any time point (p>0.05). The mean area under the curve was not significantly different between (B) control and TLR4 mutant mice (p>0.05) or (D) control and TLR4 null mice (p>0.05). Data represent means for n = 10 per group. Statistical analyses were performed using a two-way ANOVA followed by Bonferroni *post hoc* test or unpaired t-test.

To determine whether TLR4 modulates acute morphine antinociception, we compared the effects of a single submaximal antinociceptive dose of morphine in TLR4 mutant (C3H/HeJ and B10ScNJ) and control mice. Based on previous reports [17], [31], [38], we predicted that morphine antinociception would be augmented in TLR4 mutant mice. However, we found that the peak and the time course of morphine antinociception were indistinguishable between TLR4 mutant (Figure 1A) or null (Figure 1B) and their respective control mice. Specifically, no difference was observed in baseline nociceptive responses between genotypes (2.8s±0.2 vs 2.4s±0.2 for C3H/HeOuJ and C3H/HeJ, respectively, and 2.4s±0.3 vs 2.1s±0.1 for B10ScSNJ and B10ScNJ, respectively). Following morphine administration (3 mg/kg, i.p.), the maximal antinociceptive response occurred at 15–30 minutes post-injection for TLR4 mutant, null and control mice. Neither the peak morphine-induced antinociceptive responses nor the mean area under the curve (Figure 1, right panels) differed significantly between genotypes. However, there was a significant difference between mouse strains, where morphine-induced antinociception was greater in B10 strain than C3H strain mice. Statistical analysis of C3H/HeJ and C3H/HeOuJ mouse time course data using a 2-way ANOVA revealed no significant effect of treatment (F_(4,72)_ = 0.9733), but a significant effect of time (F_(4,72)_ = 11.24, p<0.0001). Statistical analysis of B10ScNJ and B10ScSNJ mouse time course data using a 2-way ANOVA revealed no significant effect of treatment (F_(4,56)_ = 0.8640), but revealed a significant effect of time (F_(4,56)_ = 33.04, p<0.0001).

### Lipopolysaccharide-induced Hyperalgesia

**Figure 2:**
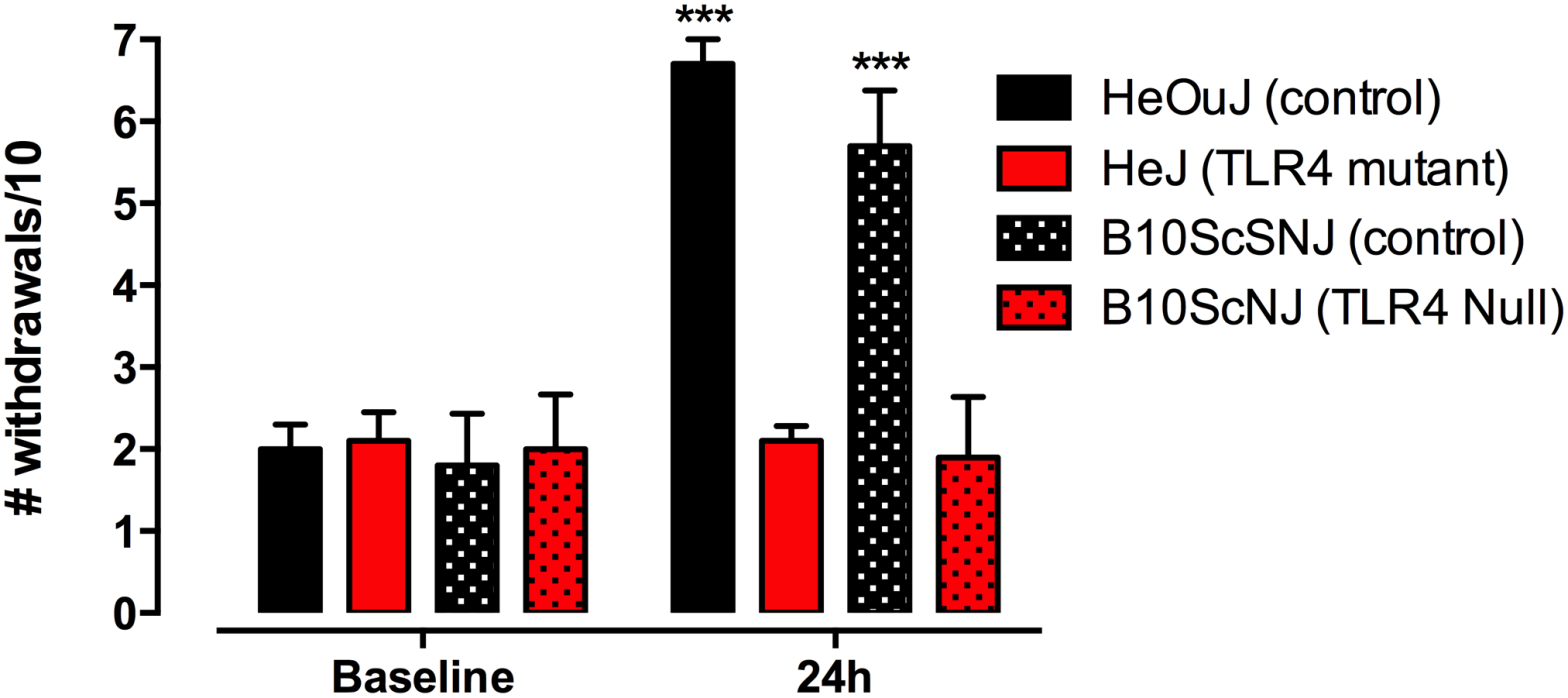
TLR4 mutant (C3H/HeJ) and null (B10ScNJ) mice are insensitive to lipopolysaccharide (LPS)-induced mechanical hyperalgesia. Mechanical thresholds in control (C3H/HeOuJ, B10ScSNJ) or TLR4 mutant/null mice were measured prior to and 24 h after LPS (1 mg/kg, i.p.) injection. No difference between baseline withdrawal thresholds was observed between genotypes. The number of paw withdrawals significantly increased in control mice 24 h post-LPS injection. No difference in mechanical sensitivity was observed between baseline and 24 h post-injection responses in TLR4 mutant mice. Data represent means for n = 10 per group. Statistical analyses were performed using a two-way ANOVA followed by Bonferroni *post hoc* test. The asterisk denotes a significant difference from baseline control response. ***p<0.001.

It is well established that systemic administration of LPS produces pain hypersensitivity. Here, we assessed the effects of LPS-induced hyperalgesia in TLR4 mutant mice. We found that baseline mechanical withdrawal thresholds did not differ between TLR4 mutant (C3H/HeJ) or null (B10ScNJ) mice and their respective controls (Figure 2). Following LPS (1 mg/kg, i.p.) treatment, a significant increase in the number of paw withdrawals to mechanical stimulation was observed in control (C3H/HeOuJ or B10ScSNJ) mice, increasing from 2.0±0.3 to 6.7±0.3, and 1.8±0.2 to 5.7±0.2 withdrawals per 10 stimulations, respectively. The number of withdrawals to mechanical stimulation following LPS administration was not different compared to baseline responses in the TLR4 mutant or null mice compared to respective controls suggesting that mechanical sensitivity was not affected by LPS administration in the mutant animals. Statistical analysis using a 2-way ANOVA revealed a significant effect of genotype (F_(3,36)_ = 40.32, p<0.0001), time (F_(3,36)_ = 155.2, p<0.0001), and interaction (F_(3,36)_ = 55.12, p<0.0001).

### Effects of Naloxone Enantiomers in Attenuating Antinociceptive Tolerance to Chronic Morphine

**Figure 3:**
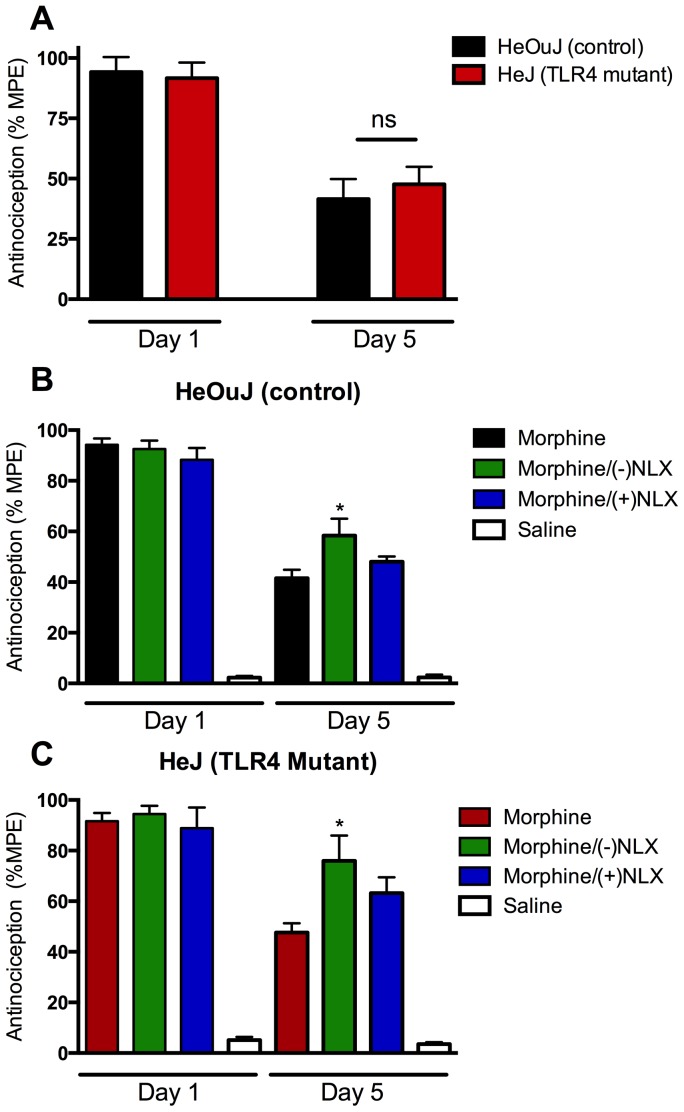
Ultra-low dose naloxone stereoselectively attenuated the loss in morphine-induced antinociception following chronic administration independent of TLR4. (A,B) Control (C3H/HeOuJ) and (A,C) TLR4 mutant (C3H/HeJ) mice were administered morphine (MS; 10 mg/kg i.p.), or MS and (−) or (+)naloxone (NLX; 1 ng/kg i.p.) once daily. Thermal nociception was measured by tail flick test 30 minutes post-injection on days 1 and 5. (A) MS-induced antinociception did not differ between TLR4 mutant or control mice over time. MS-induced antinociception was significantly reduced in both control (A,B) and TLR4 mutant (A,C) mice following chronic MS treatment. Co-treatment with (−)NLX significantly attenuated the loss in antinociception compared to MS-only treated mice in both genotypes. (+)NLX did not significantly reduce the loss of MS-induced antinociception on Day 5 compared to mice treated with MS alone in either genotype. Data represent means of n = 4–6 per group, with each n-value comprising 3–5 animals. Statistical analyses were performed using a two-way ANOVA followed by Bonferroni *post hoc* test. The asterisk denotes a significant difference from morphine-treated mice. *p<0.05.

We next examined the development of morphine analgesic tolerance in control and TLR4 mutant mice. Baseline thermal nociceptive responses were measured prior to drug administration and 30 minutes post-injection for 5 days in control (C3H/HeOuJ) and TLR4 mutant (C3H/HeJ) mice. No difference was observed in baseline thresholds between treatment groups or genotypes (Table 1). The nociceptive responses of saline-treated mice did not differ with time (Figure 3B, C), nor were there differences between genotypes (Figure 3A). As expected, daily administration of morphine significantly reduced thermal antinociceptive responses by day 5 in control mice (Figure 3A) compared to day 1 (41.5 vs 94.2% MPE), consistent with the onset of analgesic tolerance (Table 1). Unexpectedly, TLR4 mutant mice receiving morphine exhibited decreased antinociception on day 5 compared to their initial responses on day 1 (47.6 vs 91.7% MPE, Table 1, Figure 3A). The day 5 responses of morphine treated mice were not different between genotypes (Figure 3A).

**Table 1:** Mean thermal antinociceptive responses to chronic administration of morphine with or without ULD naloxone.

	Day 1		Day 3		Day 5	
Treatment	(% MPE ± s.e.m.)		(% MPE ± s.e.m.)		(% MPE ± s.e.m.)	
	control	TLR4 Mutant	control	TLR4 Mutant	control	TLR4 Mutant
SAL	2.2±0.6*	5.1±1.3*	2.9±1.3*	6.7±3.9*	2.4±1.1*	3.4±0.88*
MS	94.2±2.5	91.7±3.3	69.6±2.7	74.3±4.4	41.5±3.4	47.6±3.7
MS + (−)NLX	92.6±3.4	94.4±3.3	73.1±2.6	75.7±4.9	58.4±6.6*	76.0±9.9*
MS + (+)NLX	88.3±4.7	88.9±8.1	65.0±5.8	84.7±4.8	48.0±2.1	63.2±6.2

Control (C3H/HeOuJ) and TLR4 mutant (C3H/HeJ) mice were treated with vehicle (saline, SAL), morphine (10 mg/kg, MS), MS and (−)naloxone (1 ng/kg, NLX), or MS and (+)naloxone (1 ng/kg) once daily (i.p) for 5 days. Antinociception was measured 30 minutes post-injection by the hot-water tail flick test and are expressed as means ± s.e.m.

*significantly different compared to morphine-treated group of same genotype. Responses were not different between genotypes for any treatment at any time point.

To determine the effects of naloxone stereoisomers on morphine analgesic tolerance, mice were treated with and without (+) or (−) naloxone concomitant with morphine (10 mg/kg, i.p. for 5 days). The antinociceptive responses to drug treatments on day one were not different between treatment groups (Figure 3B, C). In control mice, the ability of ultra-low dose naloxone to attenuate the development of morphine-induced antinociceptive tolerance was stereoselective as co-administration of (−)naloxone (58.4% MPE), but not (+)naloxone (48.0% MPE) was significantly different compared to morphine-only treatment (41.5% MPE). In TLR4 mutant mice co-administration of (−)naloxone (76.0% MPE), but not (+)naloxone (63.2% MPE), was also able to significantly block the development of antinociceptive tolerance compared to morphine-only treatment (47.6% MPE). No significant difference was observed in morphine-induced antinociception at any time point between genotypes in mice chronically administered morphine, morphine and (−)naloxone or morphine and (+)naloxone.

### Opioid-induced Hyperalgesia

**Figure 4:**
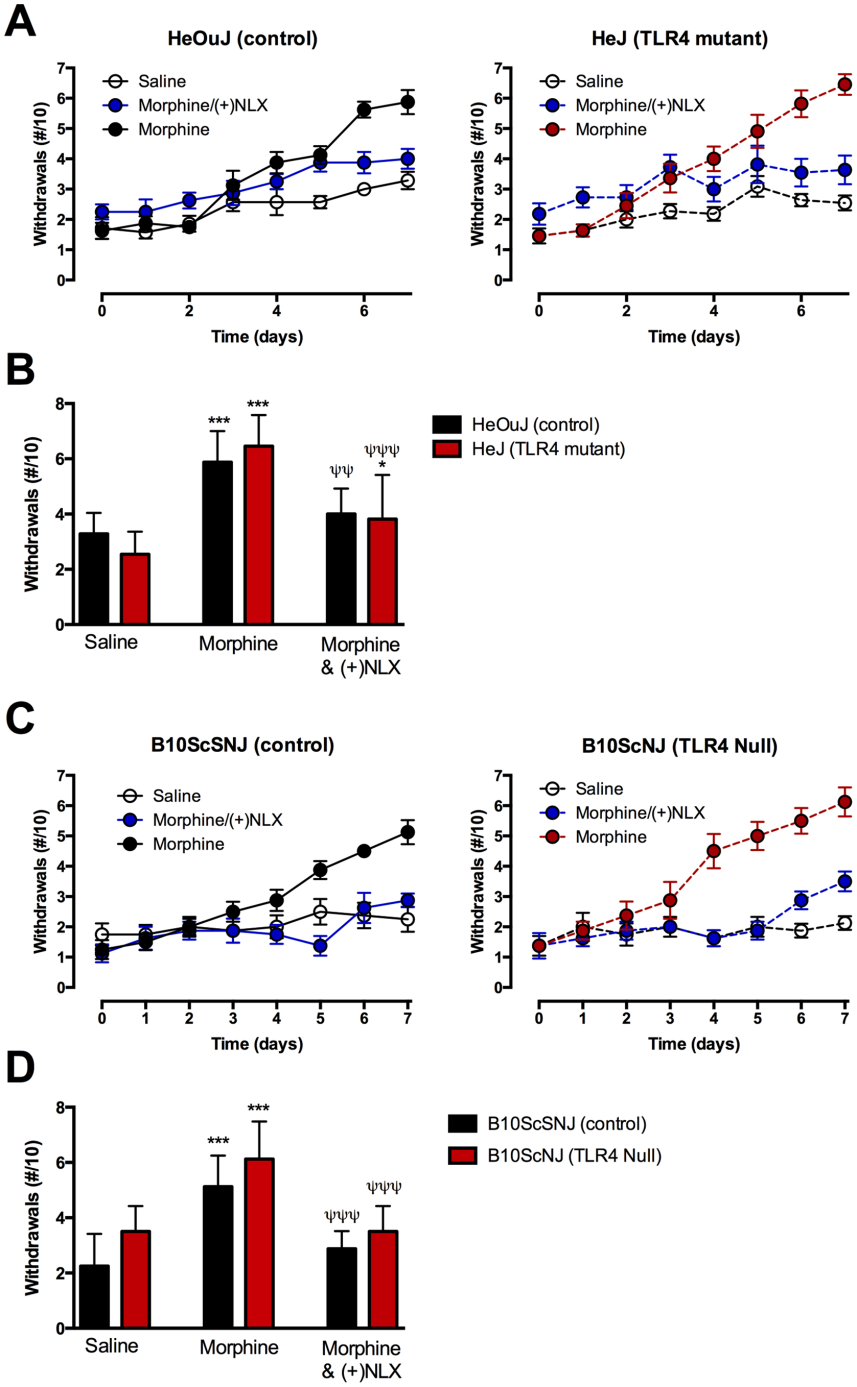
Ultra-low dose naloxone non-stereoselectively inhibited morphine-induced hyperalgesia in control and TLR4 mutant mice. (A,C) Mice were administered treatments (i.p.) twice daily, with mechanical thresholds measured daily prior to the morning injection. Number of paw withdrawals in (A) control (C3H/HeOuJ) and TLR4 mutant (C3H/HeJ) mice or (C) control (B10ScSNJ) and TLR4 null (B10ScNJ) mice treated with morphine, morphine and (+)naloxone (NLX), or vehicle (saline). A small but significant increase was observed with saline treatment over time within control and TLR4 mutant mice (p<0.01 and p<0.05, respectively) (A). (B,D) The mean number of paw withdrawals per treatment group on Day 7 in (B) TLR4 mutant (C3H/HeJ) and (D) null (B10ScNJ) mice vs respective control strains. Morphine significantly increased mechanical hyperalgesia in all genotypes. Co-administration of (−) or (+)naloxone (NLX) blocked morphine-induced hyperalgesia in control and TLR4 mutant and null mice. Data represent means for n = 7–11 per group. Statistical analyses were performed using a two-way ANOVA followed by Bonferroni post hoc test. The asterisk denotes a significant difference from saline-treated mice. *** = p<0.001. The ψ denotes a significant difference between genotypes receiving saline treatment. ^ψ ψ ψ^ = p<0.001.

Repeated escalating doses of morphine can cause a paradoxical increase in pain sensitivity (opioid induced hyperalgesia). In TLR4 mutant and TLR4 null mice, we asked whether TLR4 is critically involved in the development of opioid-induced hyperalgesia. Baseline mechanical withdrawal thresholds measured prior to drug treatment were not significantly different between control (Figure 4A, C) and TLR4 mutant (Figure 4A) or TLR4 null (Figure 4C) mice (Table 2). Control C3H/HeOuJ and B10ScSNJ mice chronically treated with escalating doses of morphine over 7 days exhibited increased mechanical sensitivity, as indicated by an increase in the number of paw withdrawals compared to saline-treated control mice (Figure 4A, C). Similarly, chronic morphine treatment also increased the number of mechanical withdrawals in the TLR4 mutant and null mice over time when compared to saline-treated animals of the same genotype (Figure 4A, C). Day 7 mechanical withdrawal responses (Figure 4B, D) are significantly increased in morphine-treated mice compared to saline controls in all genotypes (p<0.001). Morphine-induced mechanical sensitivity was not significantly different between mouse genotypes (Figure 4B, D).

**Table 2:** Effect of chronic morphine with or without ULD naloxone enantiomers on mechanical withdrawal thresholds.

	Day 0 - Baseline		Day 7	
Treatment	(# Withdrawals ± s.e.m.)		(# Withdrawals ± s.e.m.)	
	control	TLR4 Mutant	control	TLR4 Mutant
SAL	2.3±0.6	1.6±0.2	3.3±0.3* #	2.6±0.3* #
MS	1.6±0.3	1.6±0.2	5.9±0.4#	6.5±0.3#
MS +(−)NLX	1.7±0.3	1.7±0.4	3.1±0.3* #	3.2±0.2* #
MS +(+)NLX	2.3±0.3	2.6±0.3	4.0±0.3* #	3.8±0.1* #

Control (C3H/HeOuJ) and TLR4 mutant (C3H/HeJ) mice were treated with vehicle (saline, SAL), escalating morphine (10–40 mg/kg, MS), MS and (−)naloxone (1 ng/kg, NLX), or MS and (+)naloxone (1 ng/kg) twice daily (i.p) for 7 days. Mechanical withdrawal thresholds were measured prior to the morning injection and are expressed as mean number of withdrawals to 10 repeated stimulations ± s.e.m.

*significantly different compared to morphine-treated group of same genotype.

# significantly different compared to baseline (Day 0) response. Responses were not different between genotypes for any treatment at any time point.

To determine the effect of naloxone stereoisomers on opioid induced hyperalgesia in TLR4 deficient mice, animals received concomitant treatment of ultra-low dose (+) or (−)naloxone with morphine. Co-administration of ultra-low dose (+)naloxone blocked the morphine-induced increase in mechanical sensitivity in both control (Figure 4B, D) and TLR4 mutant (Figure 4B) and TLR4 null (Figure 4D) mice, where the number of withdrawals were not different from saline-treated animals. Co-administration of (−)naloxone also blocked the morphine-induced increase in mechanical thresholds in all genotypes (data not shown). Statistical analysis of TLR4 null mice using a 2-way ANOVA revealed a significant effect of treatment (F_(2,42)_ = 48.86, p<0.0001) but not of genotype (F_(1,42)_ = 2.93, p = 0.094). Statistical analysis of TLR4 mutant mice using a 2-way ANOVA revealed a significant effect of treatment (F_(2,55)_ = 43.93, p<0.0001) but not of genotype (F_(1,55)_ = 0.2157, p = 0.6442).

### Naloxone-precipitated Morphine Withdrawal and c-fos Activation

**Figure 5:**
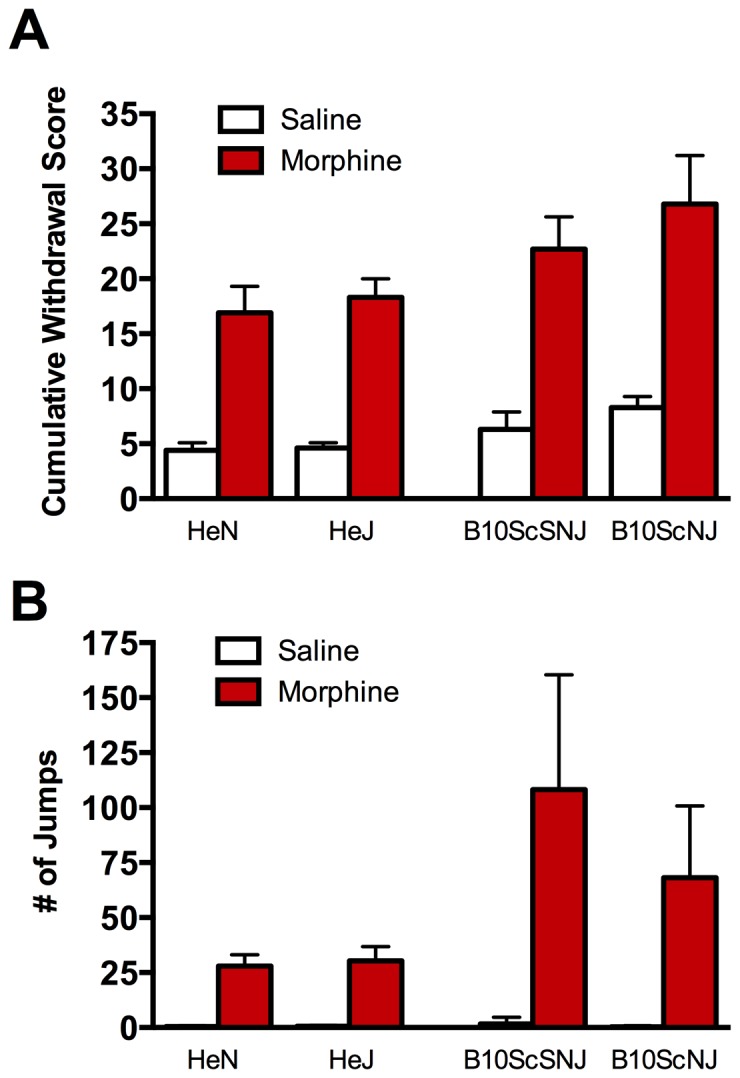
Naloxone-precipitated withdrawal syndrome in control and TLR4 mutant and null mice. (A) Withdrawal cumulative score and (B) Total number of jumps were significantly higher in both morphine-treated groups as compared with the saline controls, but no differences were detected between C3H/HeJ or B10ScNJ and their respective control strain. Data represent means for n = 5–6 per group. Statistical analyses were performed using a two-way ANOVA followed by Bonferroni post hoc test. p>0.05 for genotype.

To determine whether TLR4 is required for the development of morphine physical dependence, we first assessed the behavioural signs of naloxone-precipitated withdrawal in morphine treated TLR4 mutant and null mice. In both genotypes, naloxone evoked robust autonomic (salivation, piloerection, ejaculation) and somatic-motor responses (jumping, headshakes, wet-dog shakes, grooming, paw tremors) (Figure 5A, B). The severity of this withdrawal response – indicated by increased cumulative withdrawal scores and greater frequency of jumps – was comparable in morphine treated TLR4 mutant (C3H/HeJ) vs morphine treated control (C3H/HeN) mice. Likewise, the withdrawal response did not differ between morphine treated TLR4 null (B10ScNJ) and wild-type (B10ScSNJ) mice. Statistical analysis of cumulative withdrawal behaviours in TLR4 null mice using a 2-way ANOVA revealed a significant effect of treatment (F_(1,16)_ = 133.6, p<0.0001), but not of genotype (F_(1,16)_ = 4.08, p = 0.060) where the TLR4 nulls show withdrawal behaviours comparable to controls. Statistical analysis of cumulative withdrawal behaviours in TLR4 mutant mice using a 2 way ANOVA revealed a significant effect of treatment (F_(1,20)_ = 52.95, p<0.0001) but not of genotype (F_(1,20)_ = 0.197, p = 0.661).

**Figure 6:**
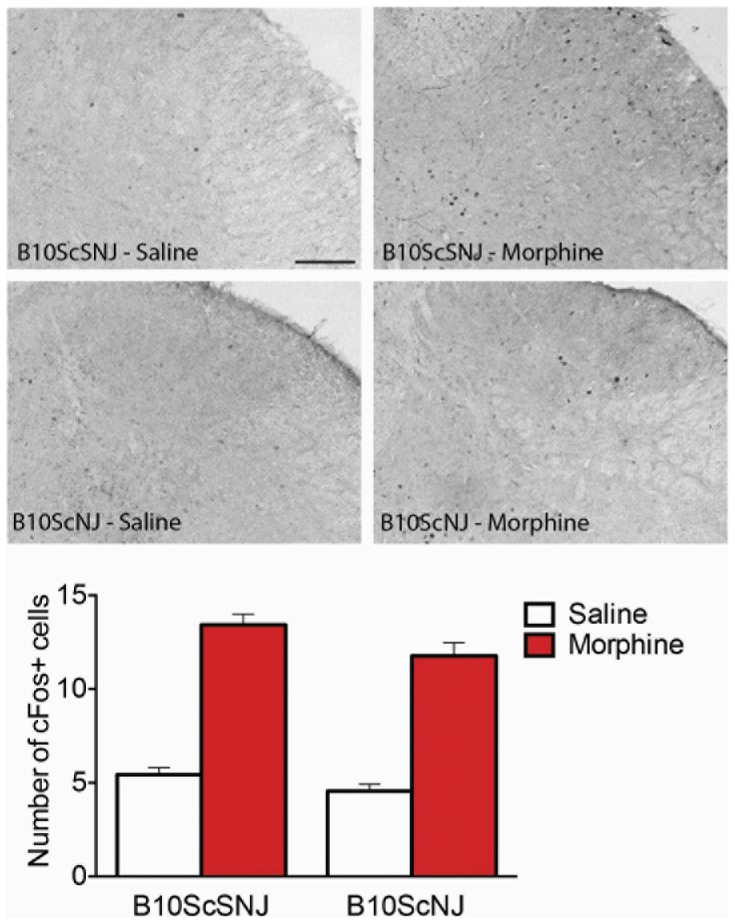
c-Fos immunohistochemical labeling in the dorsal spinal cord following naloxone precipitated withdrawal in morphine treated control and TLR null mice. Mice underwent cardiac perfusions with 4% PFA 2 h following naloxone precipitated withdrawal and spinal cords were processed for immunohistochemistry of c-fos labeling. Significant differences in labeling intensity were produced by morphine in both control (B10SCSNJ) and mutant (B10ScNJ) mice. Data represent means for n = 5–6 per group. Statistical analyses were performed using a two-way ANOVA followed by Bonferroni post hoc test. p>0.05 for genotype.

Next, we examined the expression of c-fos, which is a marker of neuronal activation; an increase in c-fos expression is a cellular correlate of opioid withdrawal. We found that naloxone significantly increased the number of c-fos positive cells in the spinal dorsal horn of both morphine treated TLR4 null and control mice (Figure 6). Statistical analysis of the number of fos positive cells in TLR4 null mice using a 2-way ANOVA revealed a significant effect of treatment (F_(1,16)_ = 32.0, p<0.0001), but not of genotype (F_(1,16)_ = 0.883, p = 0.361). Similarly, morphine-induced activation of c-fos also occurred in control C3H/HeN and TLR4 mutant (C3H/HeJ) mice but there was no effect of genotype (data not shown). Taken together, the behavioural and cellular indices of withdrawal suggest that TLR4 is not required for development of morphine physical dependence.

### Effects of TLR4 on Morphine-induced Gliosis

**Figure 7:**
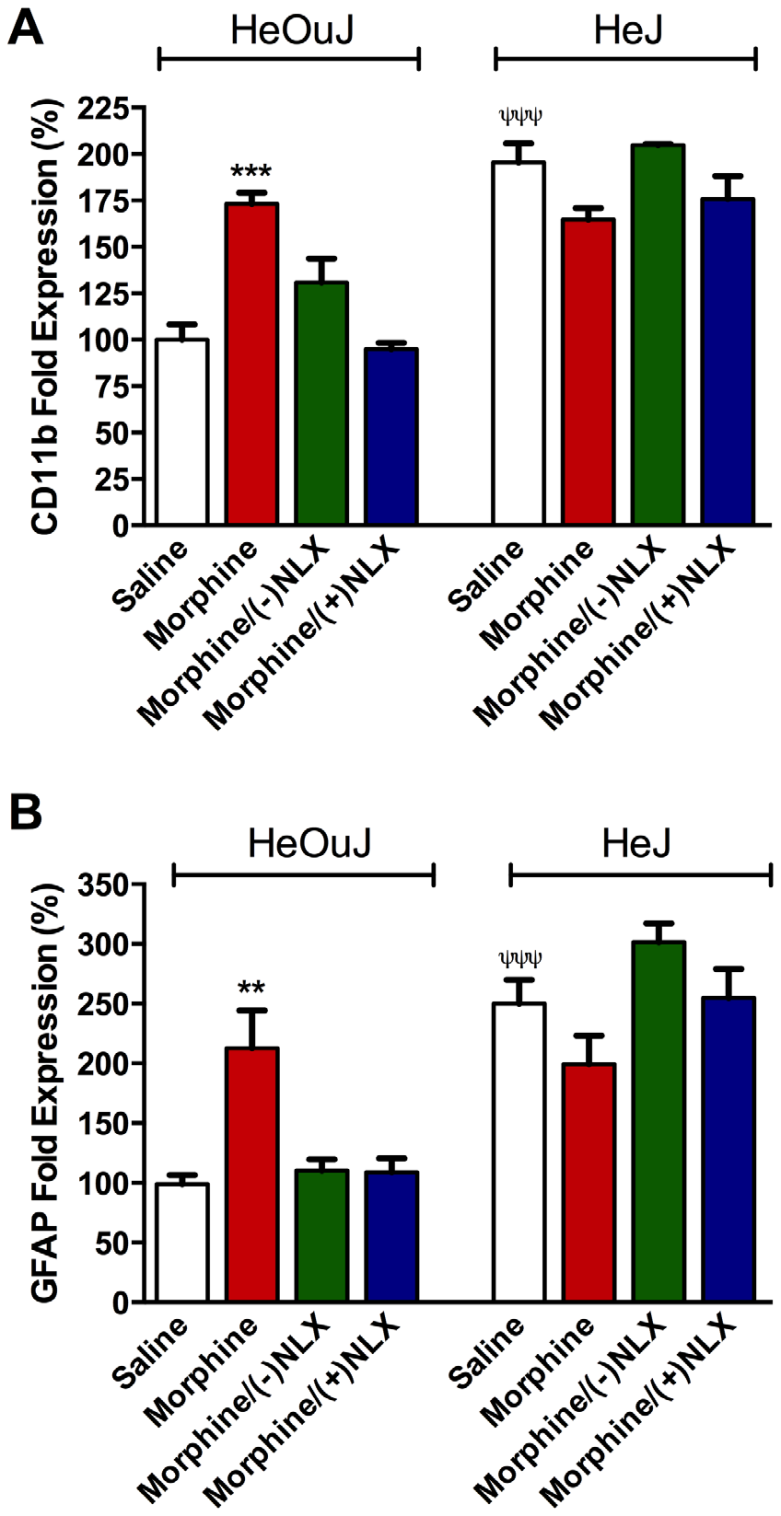
Effect of ultra-low dose naloxone and morphine treatment on CD11b and glial fibrillary acidic protein (GFAP) mRNA expression in mouse spinal cord. Wild type (C3H/HeOuJ) and TLR4 mutant (C3H/HeJ) mice were treated (i.p.) with vehicle (saline; SAL), morphine (MS; 10 mg/kg), MS and (−)naloxone (NLX; 1 ng/kg), or MS and (+)NLX (1 ng/kg), once daily for five days. Total RNA was extracted from the dorsal lumbar spinal cord, converted to cDNA templates and the expression levels of CD11b and GFAP were then assessed by quantitative real-time polymerase chain reactions as described. Values for each target gene were measured in triplicate (n = 4–5), and expressed as mean percentage expression ± s.e.m. relative to respective vehicle-treated controls (A–D) or vehicle-treated control mice (E,F). CD11b (A) and GFAP (C) mRNA expression were significantly up-regulated in MS-treated control mice; this increase was non-stereoselectively blocked by naloxone. No difference was observed between MS and (−) or (+)NLX and saline treated controls. Morphine did not significantly induce CD11b (B) or GFAP (D) mRNA up-regulation in TLR4 mutant mice, nor was there an effect of naloxone; no difference was observed between treatment groups. Significantly increased CD11b (E) and GFAP (F) mRNA expression was observed in saline treated TLR4 mutant mice compared to control mice. Data represent means for n = 5 per group. Statistical analyses were performed using a two-way ANOVA followed by Bonferroni’s *post hoc* test. The asterisk denotes a significant difference from morphine-treated mice. *P<0.05, **p<0.01, ***p<0.001. The ψ denotes a significant difference between genotypes receiving saline treatment. ^ψ ψ ψ^ = p<0.001.

To assess the role of TLR4 receptors in opioid-induced glial activation, we determined mRNA levels of CD11b (a microglial marker) and GFAP (an astrocytic marker) in the dorsal lumbar spinal cord of control and TLR4 null (B10ScNJ) mice. Animals from behaviour studies were used for this analysis. First we determined the effect of functional TLR4 on morphine-induced changes in CD11b and GFAP mRNA using tissue from mice described in section 2.7.2 (tolerance studies). Chronic morphine treatment induced a significant increase in CD11b and GFAP mRNA expression in control (C3H/HeOuJ) mice that was attenuated by co-administration of either (−) or (+)naloxone (Figure 7). Statistical analysis of CD11b mRNA expression using a 2-way ANOVA revealed a significant effect of genotype (F_(1,29)_ = 5.59, p<0.05) and treatment (F_(3,29)_ = 26.98, p<0.0001). In TLR4 mutant mice, chronic morphine treatment did not significantly alter CD11b or GFAP mRNA expression compared to saline treated mice (Figure 7). Co-treatment with (−) or (+)naloxone did not alter CD11b or GFAP mRNA expression in TLR4 mutant mice compared to saline controls. However, a genotype effect was evident when comparing the expression of CD11b and GFAP mRNA in saline-treated animals (Figure 7). A significant increase in the expression of CD11b and GFAP mRNA was observed in TLR4 mutant mice compared to saline-treated control strain mice (Figure 7, white bars).

**Figure 8:**
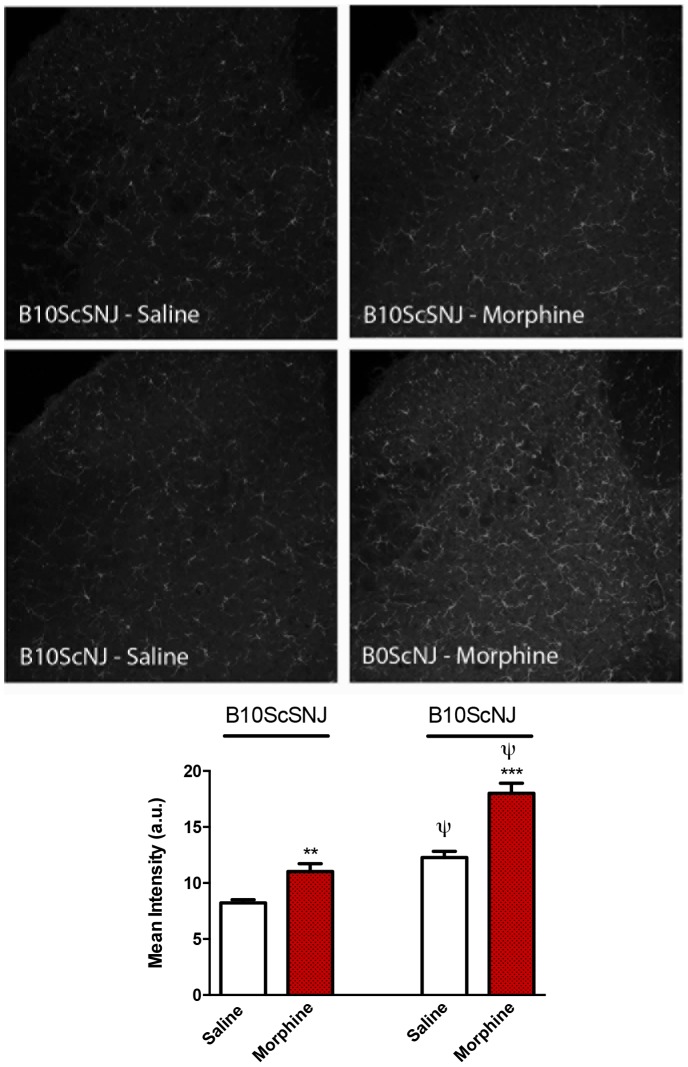
Morphine induces microglial activation in control and TLR4 null mice. Control (B10ScSNJ) and TLR4 null (B10ScNJ) mice were treated with escalating doses of morphine over 5 days. Mice were underwent cardiac perfusions with 4% PFA 2 h following naloxone precipitated withdrawal and processed for immunohistochemistry of Iba-1 labeling. Significant differences in labeling intensity were produced by morphine in both groups of mice. Additionally, a genotype effect was identified where more microglial activation was identified in the TLR4 null mouse compared to control strain. Data represent means for n = 5 per group. The asterisk denotes a significant difference from saline-treated control response. **p<0.01, ***p<0.001. The ψ denotes a significant difference between genotypes receiving saline treatment.

To determine the effects of chronic morphine treatment on microglial activation, mice from section 2.7.5 (physical dependence study) were used for immunolabeling of Iba-1. Iba1 is a 17-kDa EF hand protein that is specifically expressed in macrophages/microglia and is up-regulated during the activation of these cells. Chronic morphine treatment significantly increased Iba-1 immunolabeling in control B10 ScSNJ mice (Figure 8). Likewise, in TLR4 null mice, chronic morphine significantly increased Iba-1 immunolabeling compared to saline-treated mice (Figure 8). Similar results were obtained from C3H/HeOuJ and C3H/HeJ mice used in the opioid-hyperalgesia study (section 2.7.3, data not shown). Interestingly, a genotype effect was also evident when comparing the expression of Iba-1 labeling in TLR4 null and control strain mice treated with saline (Figure 8) where there was more microglial activation in TLR4 null mice in both saline and morphine treatment groups compared to control genotype. Statistical analysis using a 2-way ANOVA revealed a significant effect of genotype (F_(1,18)_ = 52.90, p<0.0001) and treatment (F_(1,18)_ = 67.04, p<0.0001).

## Discussion

It has been suggested that TLR4 modulates opioid analgesia and contributes to opioid-induced tolerance, hyperalgesia, and dependence [17], [28], [31], [38]. However, a recent study by Fukagawa and colleagues [39] reported a dissociation between TLR4 and opioid tolerance; they found that morphine tolerance develops in the absence of TLR4. Here, we examined the link between TLR4 and opioid analgesia in two inbred mouse strains: C3H/HeJ mice which have a dominant-negative point mutation in the *Tlr4* gene, and B10ScNJ mice which are null mutants for TLR4. Consistent with the literature, we found that morphine-induced antinociception was significantly different between mouse strains where morphine produced a greater effect in B10ScSNJ compared to C3H/HeOuJ mice [40], [41]–[44]. However, despite having nonfunctional TLR4, antinociceptive responses to a single injection of morphine in the TLR4 transgenic mice were similar to the antinociceptive responses in genotype controls. We also showed that repeated morphine treatment in TLR4 mutant strains caused: i) a loss of morphine antinociception (tolerance), ii) reduction in mechanical threshold (hyperalgesia), and iii) development of physical dependence. Taken together, our results indicate that TLR4s do not modulate acute morphine analgesia and are not necessary for the development of morphine-induced tolerance, hyperalgesia, or physical dependence.

It has been reported that acute analgesia induced by oxycodone and morphine is potentiated in TLR4 null mice compared to wild type Balb/c mice in the hot-plate paw withdrawal assay [38], [45].

However, a subsequent study by the same group reported no difference in acute analgesia produced by morphine (7.5 mg/kg) in Balb/c mice (control strain), TLR4 knockout, or MyD88 knockout mice [28]. Likewise, we found that acute morphine antinociception (measured by hot-water tail immersion test and von Frey filament test) was comparable in TLR4 mutant, TLR4 null, and wild-type control mice (background strains being C3H and B10ScSNJ). A number of methodological differences may account for these conflicting results, including the agonist, nociceptive assay and mouse genotype used. The behavioural assays used to determine opioid antinociception may contribute to the different results, as specific agonist-induced antinociception is reportedly varied between specific assays, particularly between spinal (e.g. tail flick) versus supraspinally (e.g. hot plate) mediated responses [36], [40], [46]–[50]. Also, large variations in sensitivity to opioid-induced analgesia are reported between mouse strains [40]–[44]. Indeed, our results demonstrate a significant difference in morphine-induced antinociception in C3H and B10 genetic backgrounds. The cumulative effect of these differences on acute opioid-induced analgesia is difficult to quantify; nevertheless, we found that acute morphine-induced analgesia was not different in mice lacking functional TLR4 or in TLR4 null mice compared to appropriate genotype controls.

Another key finding in our study was that opioid-induced hyperalgesia and naloxone precipitated morphine withdrawal remained intact in TLR4 mutant and null mice. These data are consistent with the report that naloxone precipitated withdrawal was not different between TLR4 knockout and control strain mice [28]. As a second line of evidence, we evaluated the effects of (+) and (−)naloxone, where both enantiomers inhibit TLR4 activity, but only the (−)isomer is an antagonist of opioid receptors. We discovered that ultra-low dose (−)naloxone attenuates morphine tolerance, whereas both (+) and (−)naloxone block opioid-induced mechanical hyperalgesia and gliosis. Our data support previous findings that opioid receptor inactive isomers of naloxone can attenuate opioid-induced hyperalgesia [13]. However, our results suggest that the (+)naloxone effect is not entirely mediated via TLR4. These data are in agreement with that of Ferrini *et al.* [13], which reported (+)naloxone blocked chronic morphine-induced thermal hyperalgesia, but not tolerance. Ferrini et al. [13] demonstrated that ultra-low dose naloxone prevents opioid hyperalgesia via two complementary microglia-dependent mechanisms: via inhibiting opioid receptor-dependent up-regulation of microglial P2×4 receptor expression and suppressing opioid receptor-independent release of brain derived neurotrophic factor (BDNF) from spinal microglia. Given that (+) naloxone is a putative ligand of TLR4 [38], the attenuation of opioid induced hyperalgesia in TLR4 null mice was unexpected. Previous studies implicating TLR4 as the target of (+)naloxone-induced effects relied primarily on binding studies [38], yet one study reported that the ability of (+)naloxone (8 mg/kg) to increase in morphine analgesia was absent in TLR4 knockout mice. However, the conclusion of this finding is confounded by a significant genotype effect, where TLR4 knockout animals had significantly higher morphine-induced analgesia compared to control strain. Therefore, the negative finding may be due to an upper ceiling effect (maximal analgesia without (+)naloxone) making it unlikely to detect a further increase by (+)naloxone.

An important consideration for comparing our results with that previously published is the observation that (+)naloxone reportedly binds to the LPS-binding pocket or MD-2, rather than TLR4 [38], and that MD-2 is required for TLR4 signalling. It is unknown whether any remaining MD-2 signalling may remain in TLR4 mutant or null mice linked to other receptors, or that the mechanism responsible for opioid-induced hyperalgesia and tolerance may be mediated via an alternative toll-like receptor. For example, TLR2 has been implicated in morphine-induced microglial activation [51]. Moreover, morphine increases expression of TLR9 in microglia that is dependent on functional mu opioid receptors [52]. Perhaps most relevant to the discussion is the recent observation that opioid agonists such as morphine and fentanyl produce only weak activation of TLR4 signalling where 3 and 10 µM, but not 20 and 100 µM morphine were shown to activate TLR4 and 0.3 µM but not 1–100 µM fentanyl activated TLR4 [53]. Additionally, morphine was shown to inhibit (not potentiate) LPS-induced TLR4 activity via a non-G-protein coupled receptor [53]. Considering lack of TLR4 involvement in opioid-induced effects in the present study, it was important to confirm that TLR4 function was abrogated in the transgenic mice. Activation of TLR4 with LPS has been shown to cause robust hyperalgesia [35], [54], therefore, we reasoned that in the absence of functional TLR4s, LPS-induced hyperalgesia would be abrogated. Indeed, we found that LPS injection had no effect on nociceptive threshold in TLR4 mutant and null mice, whereas wild-type control mice displayed significant LPS-induced hyperalgesia. Our findings are consistent with Hoshino *et al.* [33], who concluded that TLR4 mutant and null mice are LPS-insensitive and lack LPS-induced signalling.

It is widely recognized that spinal gliosis is associated with analgesic tolerance following chronic opioid administration, but is not observed following acute opioid exposure [16], [55]. We have previously shown that co-administration of ultra-low dose naltrexone inhibited glial activation, in addition to attenuating tolerance, following chronic morphine treatment [32]. In the present study, naloxone non-stereoselectively inhibited activation of spinal microglia and astrocytes, indicated by the attenuation of morphine-induced CD11b and GFAP mRNA (markers indicative of activated microglia and astrocytes, respectively) expression; however, these cellular effects do not correlate with the stereoselective inhibition of antinociceptive tolerance by (−)naloxone. In agreement, Ferrini et al [13] also reported (+)naloxone blocked morphine-induced activation of spinal microglia without preventing analgesic tolerance in rats. One potential mechanism for these observations is that ultra-low dose opioid antagonists target non-opioid glial receptors, such as TLRs, which have been shown to bind levo and dextro opioid enantiomers [17], [26]. Here we showed that the development of opioid tolerance remains intact in TLR4 mutant and null mice. Thus, we conclude that TLR4 receptors are not required for tolerance and they are likely not the target of ultra-low dose (+)naloxone. In agreement, Fukagawa and colleagues [39] reported no difference in the development of morphine tolerance in TLR4 mutant C3H/HeJ and control C3H/HeN mice, nor between wild type (C57BL/6J) and TLR4^−/−^ mice. Interestingly, morphine did not induce up-regulation of CD11b or GFAP in the spinal cord of TLR4 mutant mice compared to saline-treated controls. This is in contrast to a previous report in which chronic morphine was found to cause increased CD11b expression in TLR4^−/−^ mice compared to controls [39]. The sizable difference in morphine dose (10 mg/kg vs 60 mg/kg in the Fukagawa study) and the different mouse strains employed may explain the discord. Importantly, in the present study we report a significant increase in CD11b and GFAP mRNA expression and Iba-1 immunolabeling in vehicle-treated TLR4 mutant and null mice compared to vehicle treated control mice indicating a genotype effect without pharmacological treatment. These data are consistent with increased macrophage and astrocyte labeling in TLR4 mutant mice compared to control mice [24]. Activation of innate immunity via TLR4 is essential for pathogen recognition and host defense. In the absence of pathogens it is not known how the innate immune system is altered by the absence of TLR4. It has been suggested that TLR4 deficient mice (C3H/HeJ mice) exhibit *enhanced* spinal neuro-inflammation and gliosis compared to wild type control following spinal cord injury, which correlated with significant impairments of normal progression of spinal cord injury repair and functional recovery in TLR4 deficient mice [24]. Given the immune-compromised state of these TLR4 mutant and null mice, their glia may be in a state of reactivity rather than the normal resting state found in control strains; however, it is interesting that this glial activation did not affect baseline nociceptive thresholds. We suggest that opioid-induced gliosis was masked by an already alerted immune system in the mutant and null mice.

## Conclusion

The most parsimonious interpretation for our findings is that TLR4 is not required for the development of morphine-induced analgesic tolerance, hyperalgesia, or physical dependence. This conclusion is based on the occurrence of these phenomena in two independent TLR4 mutant and null transgenic mouse lines. In addition, our data suggest TLR4s do not mediate the effects of ultra-low dose (−) or (+) naloxone as treatment with these stereoisomers remained effective in TLR4 null and mutant mice.
