# Correction: Toll-Like Receptor 4 Mutant and Null Mice Retain Morphine-Induced Tolerance, Hyperalgesia, and Physical Dependence

**DOI:** 10.1371/journal.pone.0101621

**Published:** 2014-06-24

**Authors:** The PLOS ONE Staff

## Notice of Republication

This article was republished on June 3rd, 2014, due to the figures being ordered incorrectly in the previously published article. Additionally, the name of the fourth author now reads Christopher J. B. Nicol. Please download the PDF again to view the corrected article. The originally published, uncorrected article and the republished, corrected article are provided here for reference.

## Supporting Information

File S1 Originally published, uncorrected article.
(PDF)

File S2 Republished corrected article.
(PDF)

## References

[1] Mattioli TA, Leduc-Pessah H, Skelhorne-Gross G, Nicol CJB, Milne B, et al (2014) Toll-Like Receptor 4 Mutant and Null Mice Retain Morphine-Induced Tolerance, Hyperalgesia, and Physical Dependence. PLoS ONE 9(5): e97361 doi:10.1371/journal.pone.0097361 24824631 10.1371/journal.pone.0097361 PMC4019634

